# Chemical Characterization and Quantification of Titanium Dioxide Nanoparticles (TiO_2_-NPs) in Seafood by Single-Particle ICP-MS: Assessment of Dietary Exposure

**DOI:** 10.3390/ijerph17249547

**Published:** 2020-12-20

**Authors:** Alfina Grasso, Margherita Ferrante, Pietro Zuccarello, Tommaso Filippini, Giovanni Arena, Maria Fiore, Antonio Cristaldi, Gea Oliveri Conti, Chiara Copat

**Affiliations:** 1 Department of Medical, Surgical and Advanced Technologies “G.F. Ingrassia”, University of Catania, Via Santa Sofia 87, 95123 Catania, Italy; agrasso@unict.it (A.G.); pietro.zuccarello@unict.it (P.Z.); giovanniare@yahoo.it (G.A.); mfiore@unict.it (M.F.); antonio.cristaldi@unict.it (A.C.); olivericonti@unict.it (G.O.C.); ccopat@unict.it (C.C.); 2 Environmental, Genetic and Nutritional Epidemiology Research Center, Department of Biomedical, Metabolic and Neural Sciences, Section of Public Health, University of Modena and Reggio Emilia, Via Campi 287, 41125 Modena, Italy; tommaso.filippini@unimore.it

**Keywords:** titanium dioxide, nanoparticle, *sp*ICP-MS, processed food, dietary intake, E171

## Abstract

The significant increase in the production and variety of nanoparticles (NPs) has led to their release into the environment, especially into the marine environment. Titanium dioxide nanoparticles (TiO_2_-NPs) are used in different industrial sectors, from the food industry to several consumer and household products. Since the aquatic environment is highly sensitive to contamination by TiO_2_-NPs, this work aimed to give a preliminary assessment of the contamination of packaged seafood, where the food additive TiO_2_ (E171) is not to be intentionally added. This allowed providing a chemical characterization and quantification of TiO_2_-NPs in processed canned fish products belonging to different trophic positions of the pelagic compartment and in canned clam. The new emerging technique called single-particle inductively coupled plasma mass spectrometry (*sp*ICP-MS) was applied, which allows the determination of nanoparticle number-based concentration, as well as the dissolved titanium. This study highlights how processed food, where the pigment E171 was not intentionally added, contains TiO_2_ in its nanoparticle form, as well as dissolved titanium. Processed clam represented the seafood with the highest content of TiO_2_-NPs. In pelagic fish species, we found progressively higher levels and smaller sizes of TiO_2_-NPs from smaller to larger fish. Our results highlight the importance of planning the characterization and quantification of TiO_2_-NPs in food both processed and not, as well as where the pigment E171 is intentionally added and not, as it is not the only source of TiO_2_-NPs. This result represents a solid step toward being able to estimate the real level of dietary exposure to TiO_2_-NPs for the general population and the related health risks.

## 1. Introduction

In Earth science, nanoparticles (1–100 nm) are important components of the biogeochemical system, but human impact on the environment has altered their natural cycle [1] by introducing engineered nanoparticles with several physicochemical characteristics and applications [2]. Nanoparticles represents a blooming industrial sector destined to increase because of the numerous investments that it is able to attract. Due to their peculiar characteristics (e.g., different optical properties, greater flexibility, resistance, reactivity, electrical conductivity, or absorption), nanoparticles (NPs) are finding more and more applications in many consumer products, such as conductors, cosmetics, plastics, and agents used in environmental recovery, as well as in various sectors such as the pharmaceutical, food, biomedical, military, and automotive industries [3,4].

Our study focuses on titanium dioxide (TiO_2_), a metal oxide naturally occurring in three polymorphic forms known as rutile, anatase, and brookite [2]. It is synthetized as titanium dioxide nanoparticles (TiO_2_-NPs) for several industrial sectors. In the food industry, TiO_2_ is authorized as a food additive (E 171) in accordance with the European Regulation (EC) No. 1333/2008, in both anatase and rutile forms (Commission Regulation (EU) No. 231/2012) [5]. It is added for whitening and brightening purposes in many foods including milk and dairy products, chewing gum, ice cream, and all sweets where it is included in the coating of sugar confectionary [5,6]. Nevertheless, it is not considered a nanomaterial by the current “European Commission recommendation for the definition of nanomaterials”. Accordingly, a nanomaterial should consist for 50% or more of particles having a size between 1 and 100 nm (Regulation 211/696/EU), but E171 may contain up to 3.2% of its weight in nanoparticles. TiO_2_ in its nanoform is also used in plastic packaging, as titanium dioxide surface-treated with fluoride-modified alumina, to improve the quality and the shelf-life of products. According to the European Food Safety Authority (EFSA) safety assessment, the substance does not raise safety concerns for the consumer if used as an additive up to 25% *w*/*w* in polymers in contact with all food types in any time and temperature conditions [7]. TiO_2_-NPs are also used in several consumer and household products to which humans can be potentially exposed such as toothpaste, sun cream, creams, and lip balm [8,9,10]. Other uses of the manufacturer TiO_2_ nanomaterial known as P25 include antimicrobial applications, paints, catalysts for air and water purification, medical applications, and energy storage [11]. Despite their favorable properties, NPs are known to cause local adverse effects and/or systemic toxicity [12].

The significant increase in the production and variety of nanomaterials has led to their release into the environment [13], especially into the marine environment, as the NPs are not completely removed from domestic and industrial wastes after water treatment [14,15]. This increases both the ecological risks for the ecosystems and the likelihood of inhalation or dermal and oral exposure for humans [16]. In toxicological studies concerning TiO_2_-NPs, most researchers utilized the industrial-grade nanoform of TiO_2_, although food grade one represents the majority of TiO_2_-containing materials that enter the ecosystem today [17]. The choice depends on the fact that it is commonly used, due to the primary crystals being relatively uniform and less than 50 nm in size. Studies also demonstrated a difference in the removal efficiency of food-grade TiO_2_, which is lower than industrial-grade TiO_2_. For this reason, food-grade TiO_2_ accumulates preferably in the environment, involving an environmental and human health risk [17].

The oral exposure route of TiO_2_-NPs has been the least investigated. To date, despite the existing numerous applications of TiO_2_-NPs, little or nothing is known about the bioaccumulation potential of TiO_2_-NPs along the food chain and the dietary exposure dose to the general population. Nevertheless, as this compound has a limited elimination rate and as it can be absorbed by the gastrointestinal tract in a size-dependent manner and pass through the mucus pores to enter other organs [18], some studies suggested a potential health risk such as disorders of the gut microbiota and gut-associated metabolism in vivo [19], apoptosis induction [20], hepatic toxicity [21], a potential risk for liver, ovaries, and testes [22], an impact on lipid metabolism [23], and an increased abundance of proinflammatory immune cells and cytokines in the colonic mucosa [24].

The risk to humans is through food chain transport because TiO_2_ has a high tendency to bioaccumulate in aquatic organisms, and this implies that TiO_2_ particles could be transferred from one species to another [17].

Since the aquatic environment is highly sensitive to contamination by TiO_2_-NPs [13], this work aimed to give a preliminary assessment of the contamination of packaged food derived from marine environment. According to the literature, very few studies reported the levels of nanoparticles in marine organisms [25,26,27,28,29].

With this study, we wanted to provide a chemical characterization and quantification of TiO_2_-NPs (<100 nm), TiO_2_ total particles (TiO_2_-Ps-Tot, all particle sizes), dissolved Ti, and total Ti in processed canned seafood products belonging to different trophic positions. Our choice to use canned seafood for this evaluation study was because it is a fundamental component in human nutrition, representing an important source of unsaturated fatty acids, protein, and different micronutrients [30].

Furthermore, we also provided data related to the estimated daily intake for both adults and children, which may be useful to risk assessors for developing a provisional tolerable daily intake for TiO_2_-NPs.

## 2. Methods

### 2.1. Alkaline Digestion and Single-Particle Inductively Coupled Plasma Mass Spectrometry (spICP-MS) Analysis

Different brands of canned tuna (five), mackerel (four), anchovy (three), and clam (three), among the best-selling and low-cost brands, were purchased from the main Italian supermarket chains in the city of Catania, (Italy), in the period between June and July 2019, and stored at −80 °C until analysis. For each brand of seafood product, we also chose to purchase three different batches, which were extracted and processed in triplicate. Thus, we performed a total of 45 extractions for canned tuna, 36 for canned mackerel, 27 for canned anchovy, and 37 for canned clam.

Assessment of TiO_2_ was performed using a NexION^®^ 350D (Perkin Elmer, Waltham, MA, USA) with the Syngistix Nano Application software (Perkin Elmer, Waltham, MA, USA). The instrumental operating conditions for the determination of TiO_2_ are listed in Table 1. The dwell time used was chosen on the basis of other studies [27,31].

The new emerging technique of single-particle inductively coupled plasma mass spectrometry (*sp*ICP-MS) allows the determination of particle number-based concentration, with rapid simultaneous characterization of the elemental composition, number of particles, size and size distribution, and dissolved concentration. Furthermore, by modifying the integration window, it is possible to collect data related to a specific size distribution. Accordingly, for each sample, we captured data on the total TiO_2_ particles (Ps-Tot) and TiO_2_ nanoparticles (NPs < 100 nm) only.

A titanium nanoparticle stock solution was prepared from a TiO_2_-NP standard (60 nm TiO_2_ nanopowder, rutile, 99.9%, AEM) purchased from Nanovision (Brugherio, MB, Italy), while a Ti ion standard (1000 mg/L, CPAchem) was used for the *sp*ICP-MS calibration of titanium.

To support the quality of *sp*ICP-MS measurements, the particle size distribution of the powder TiO_2_-NPs standard was assessed as follows: a TiO_2_-NP stock suspension (215 ng/L or 4.5 × 10^5^ particles/mL) was prepared in ultrapure water and dispersed for 30 min at 37 °C using an ultrasonic bath, to maximize a homogeneous dispersion. The transport efficiency (TE%) was calculated with the certified reference material PELCO (Ag-NPs, 39 ± 5 nm, 110 ng/L, monitoring *m/z* 107) under the same instrumental conditions as the samples, obtaining a value of TE% 2.54. This solution gave a TiO_2_-NP concentration of 4.6 × 10^5^ ± 0.16 × 10^5^ particles/mL (*n* = 10), in agreement with the particle concentration prepared. TiO_2_-NPs showed a size range of 44–85 nm with a mean size of 66.2 ± 3.0 (*n* = 10) and a modal size of 56.6 ± 4.4 (*n* = 10). The results obtained were in compliance with the size of the TiO_2_-NP standard supplied by the manufacturer (60 nm).

Before performing the *sp*ICP-MS analysis, an alkaline digestion of the samples was performed using the method described by Gray et al. (2013) [32]. Approximately 0.5 g of wet sample tissue was weighed in DigiTUBEs (SCP Science, Baie D’Urfé, Québec, Canada) and mixed with 5 mL of tetramethylammonium hydroxide (TMAH, 20% *v*/*v*), which is a strong organic base capable of digesting tissues and releasing nanoparticles without altering them. A vortex was used, at first, to prevent the tissues from sticking to the walls of the container used for digestion. The TiO_2_ extraction was obtained through sonication for 30 min at 37 °C using an ultrasonic bath to accelerate tissue breakdown and prevent particle aggregation. Subsequently, the samples were left to digest for another 24 h at room temperature. At the end of digestion, samples were diluted with MilliQ water (Millipore, Bedford, MA, USA) to 1% TMAH concentration before analysis and 0.1% Triton X-100 to allow the detection of single particles.

All samples and calibration solutions were sonicated for 30 min before being analyzed. To avoid contamination between samples, the system was rinsed with nitric acid (2%, *v*/*v*) prior to the measurement. The TiO_2_-NP standard was also used for the determination of the transport efficiency within the 3–8% range, in agreement with the TE obtained with an Ag certified reference material.

The effect of the extracting solution on the size distribution and particle concentration was studied in triplicate, simultaneously evaluating the TiO_2_ standard in ultrapure water (n_1_ = 3) and TMAH 1% (n_2_ = 3), at the same concentration (215 ng/L or 4.5 × 10^5^ particles/mL) we used for determining the transport efficiency. We obtained a particle concentration of 4.3 × 10^5^ ± 5.0 × 10^4^ in water and 4.9 × 10^5^ ± 3.6 × 10^4^ in TMAH 1%, with recoveries of 97.6% ± 10.5% and 108.8% ± 7.2%, respectively. In addition, the results revealed that the alkaline digestion in TMAH 1% did not affect the TiO_2_ particle size distribution (mean size and modal size). The mean size of TiO_2_ in ultrapure water (63.8 nm ± 3.0) and mean size of TiO_2_ in TMAH 1% (61.1 nm ± 4.3) were statistically homogeneous according to a two tailed *t*-test (*p* = 95%; n1 + n2 − 2) (t_calculated_ = 1.5 < t_tabulated_ = 2.8). Moreover, the modal size was statistically homogeneous in ultrapure water (57.6 ± 3.2) and in TMAH 1% (56.0 ± 4.0) (t_calculated_ = 0.88; t_tabulated_ = 2.8).

The limit of detection (LOD) and the limit of quantification (LOQ) were calculated by analyzing 10 alkaline extract blanks, in the same analytical condition of the samples, on the basis of the mean ± 3 SD and the mean ± 10 SD criteria of the number of particles/mL obtained, respectively. The LOD was 1.3 × 10^3^ particles/mL, while the LOQ was 2.5 × 10^3^ particles/mL. Referring to the sample weight and digestion volume used, these values were equivalent to 2.6 × 10^5^/g and 5.0 × 10^5^/g, respectively.

In addition, the LOD in size (LODnm) was estimated at 35 nm using Equation (1) [26,33].

(1) $$\mathrm{LODnm}=\sqrt[{3}]{\frac{6\times 3\sigma_{blank}}{R\times f_{a}\times \rho \times \pi }},$$

where $3\sigma_{blank}$ is three times the standard deviation of the counts/dwell time of alkaline blanks (1% TMAH), *R* is the slope of the calibration curve of ionic Ti solutions, *f_a_* is the mass fraction of the analyzed metallic element in the TiO_2_ NPs, and *ρ* is the density of the TiO_2_ NPs.

For each batch of analysis, a quality control was performed with analytical recovery before and after spiking with 60 nm TiO_2_-NPs at a concentration of 5 µg/L, corresponding to a concentration of 1.1 × 10^6^ parts/mL. The values (range 90–95%) were calculated for the whole size distribution by dividing the TiO_2_-NP concentration by the TiO_2_-NP concentration found in the solution of TiO_2_-NPs used for spiking and multiplying by 100.

Accuracy was tested using a laboratory-fortified matrix (LFM) with a seafood sample spiked with 5 µg/L of TiO_2_-NPs (60 nm). An LFM was processed at each batch of digestion, obtaining a concentration of 9.8 × 10^5^ ± 4.6 × 10^5^ particles/mL (1.9 × 10^8^ ± 9.3 × 10^7^ particles/g) and a recovery range of 87–121% (*n* = 5). The measured mean size of TiO_2_-NPs in alkaline digested samples was 64.2 ± 5.1 nm.

### 2.2. Acid Digestion and ICP-MS Analysis of Total Ti

For the determination of total titanium, the same samples were processed by acid digestion. Aliquots of 0.5 g of wet samples were weighed in Teflon reactors using an analytical balance (Mettler Toledo) and then digested in a microwave oven (Ethos, TC, Milestone, Sorisole (BG), Italy), by adding 6 mL of 67% super-pure nitric acid (HNO_3_; Carlo Erba, Italy) and 2 mL of 30% hydrogen peroxide (H_2_O_2_; Carlo Erba, Italy) for 1 h at 80 °C. After acid digestion, all samples were diluted to 50 mL with ultrapure water and were filtrated through a 0.45 µm membrane filter before analysis. Total Ti was quantified with the same inductively coupled plasma mass spectrometer (ICP-MS NexION^®^ 350D, Perkin Elmer, Waltham, MA, USA) in standard mode, using the standard addition technique covering the concentration from 0 to 50 µg/L. Instrumental condition for Total Ti determination are showed in Table 2.

A single-element standard solution of Ti (1000 mg/L in 5% di HNO_3_, 0.5% HF) was purchased from CPAchem. Standards for instrument calibration were prepared in the same acid matrix, and yttrium (Y) was used as an internal standard to verify the accuracy.

To verify if acid digestion of the samples allowed the total dissolution of TiO_2_ particles and then the quantification of total Ti, we conducted a preliminary acid digestion of 4 µg/L ionic Ti (*n* = 6), canned clam (*n* = 6), and canned tuna (*n* = 6). The quantification of TiO_2_ particles was detected using Syngistix Nano Application software, and the resulting background signals, expressed as particles/mL, are shown in Table 3.

The threshold value of TiO_2_ particles was estimated as the mean + 3 SD given by ionic Ti, which resulted in 1029 particles/mL. Digested samples of canned clam and canned tuna did not show background values of TiO_2_ particles higher than the calculated threshold, demonstrating the effectiveness of the acid digestion in dissolving all TiO_2_ particles.

The LOD and LOQ were calculated by analyzing 10 acid extract blanks according to the mean ± 3 SD and mean ± 10 SD criteria, respectively. They resulted 0.06 and 0.16 mg/kg, respectively.

For each batch of analysis, a quality control was performed with analytical recovery after spiking with 20 µg/L of ionic Ti.

Accuracy was tested using a laboratory-fortified matrix with a seafood sample (use of ionic Ti at 20 µg/L) processed at each batch of digestion, obtaining a recovery range of ionic Ti from 92–115%.

### 2.3. Estimated Meal Intake

An exposure assessment derived from the consumption of selected seafood products was conducted for TiO_2_-NPs (TiO_2_ particles <100 nm), TiO_2_-Ps-Tot (TiO_2_ total particles) and dissolved Ti according to the method described in a previous paper [34]. Briefly, the estimated meal intake (EDI) (mg/kg body weight (BW) per day) was determined according to Equation (2).

EMI = (C × M)/BW,(2)

where C is the TiO_2_-NPs, TiO_2_-Ps-Tot, or dissolved Ti (mg/kg wet weight), M is the meal size (0.227 kg for adults and 0.114 kg for children) [34], and BW is the body weight, considered as 16 kg for children (6 years) and 70 kg for adults (70 years) [35].

### 2.4. Statistical Analysis

The statistical software package IBM SPSS 20.0 (IBM, Armonk, NY, USA) was used for statistical analysis. One-way ANOVA and a post hoc Tukey test were performed to evaluate differences in TiO_2_ and dissolved Ti levels among canned products. We processed a total of 45 samples of canned tuna, 36 of canned mackerel, 27 of canned anchovy, and 37 of canned clam.

## 3. Results

**Figure 1:**
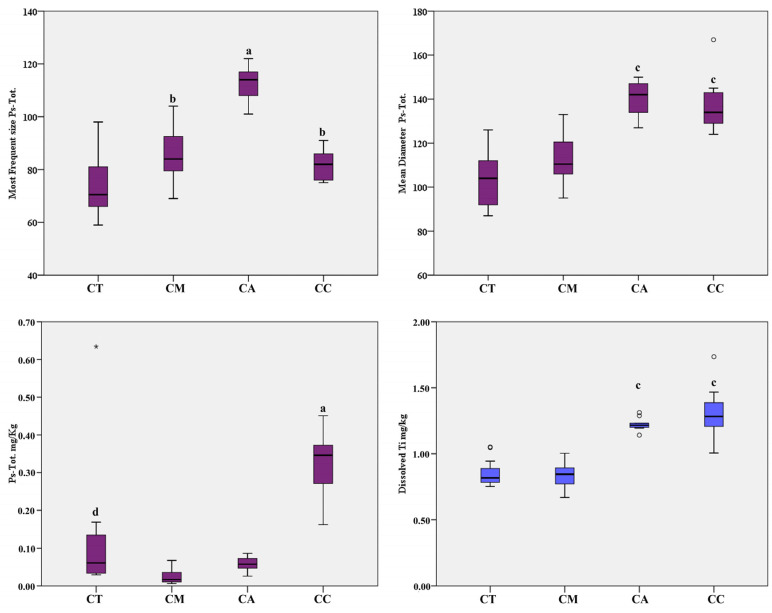
Box-plot distribution of TiO_2_-Ps-Tot modal size (nm), mean diameter (nm), level of Ps-Tot, and dissolved Ti (mg/kg) in packaged seafood products. Legend: CT, canned tuna; CM, canned mackerel; CA, canned anchovy; CC, canned clam; a, *p* < 0.001 vs. all; b, *p* < 0.05 vs. CT; c, *p* < 0.001 vs. CT and CM; d *p* < 0.05 vs. CM and CA. ° Outliers values of the distribution. * Extreme values of the distribution.

The total TiO_2_ particles present (Ps-Tot size), as well as the level of Ti in its dissolved form, were analyzed using the NexION^®^ 350D (Perkin Elmer, Waltham, MA, USA) with the Syngistix Nano Application software, by processing the samples with ultrasound-assisted alkaline digestion. In a separate working session, we also determined the level of total Ti measured with ICP-MS in standard mode, by processing the samples digested with acid. As shown in Table 4, the quantification of dissolved Ti did not differ significantly from the total Ti. The slightly higher result of total Ti can be related to the acid dissolution of the Ti bonded in the particles, as supported in the experimental data shown in Table 3. As shown in Table 4 and Figure 1, canned anchovy and canned clam revealed the highest mean diameters of Ps-Tot size, close to 140 nm, significantly higher than the mean diameters found in canned tuna and canned mackerel, close to 100 nm. The modal size was found to be significantly higher in canned anchovy versus the other seafood products, followed by canned mackerel, canned clam, and canned tuna. With regard to the Ps-Tot level, the samples of canned clam had the highest levels of TiO_2_-NPs compared to the fish species, with a mean value of 0.326 mg/kg. Among fish species, canned tuna had levels of TiO_2_-NPs significantly higher than canned mackerel and anchovy, with a mean value of 0.117 mg/kg. Considering the ionic Ti determined using the single-particle technique, canned clam and canned anchovy had higher levels than the other seafood products, with mean values of 1.31 and 1.22 mg/kg, respectively.

**Figure 2:**
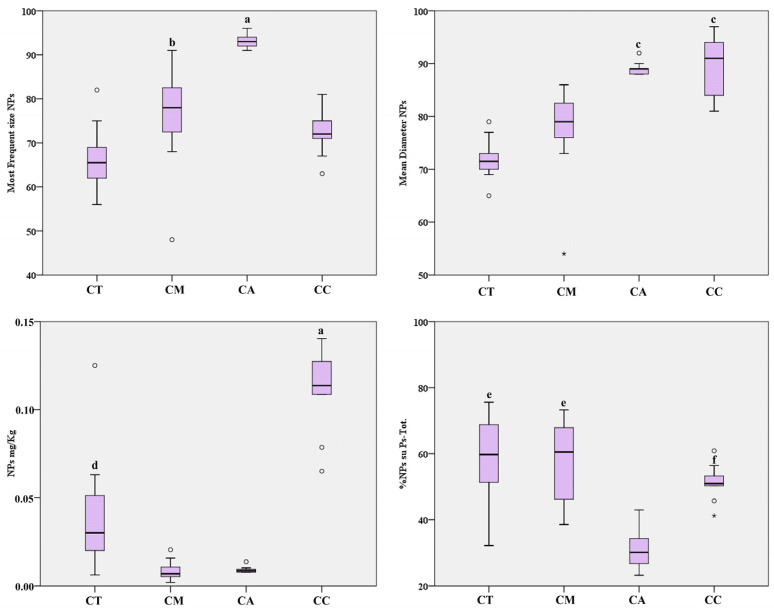
Box-plot distribution of TiO_2_-NP modal size (nm), mean diameter (nm), %NPs in Ps-Tot, and level (mg/kg) in packaged seafood products. Legend: CT, canned tuna; CM, canned mackerel; CA, canned anchovy; CC, canned clam; a, *p* < 0.001 vs. all; b, *p* < 0.01 vs. CT; c, *p* < 0.001 vs. CT and CM; d, *p* < 0.01 vs. CM and CA; e, *p* < 0.001 vs. CA; f, *p* < 0.01 vs. CA. ° Outliers values of the distribution. * Extreme values of the distribution.

For each sample, by reducing the integration window of particle size, we selected the particle size distribution <100 nm to quantify the nanoparticles only. The analyses carried out showed the presence of TiO_2_-NPs, with a diameter lower than 100 nm, in all the seafood samples. As shown in Table 5 and Figure 2, with regard to NPs size, canned anchovy and canned clam revealed the highest mean diameters, close to 90 nm, higher than the mean diameters found in canned tuna and canned mackerel (both below 80 nm). The modal size was found to be significantly higher in canned anchovy versus the other seafood products with a mean value of 93 nm, followed by canned mackerel, canned clam, and canned tuna. With regard to the NP level, the samples of canned clam had the highest levels of TiO_2_-NPs compared to the fish species, with a mean value of 0.112 mg/kg. Among fish species, canned tuna had levels of TiO_2_-NPs fourfold higher than canned mackerel and anchovy, with a mean value of 0.038 mg/kg. Furthermore, the percentage of TiO_2_-NPs in the TiO_2_ total particles was close to 60% in all seafood products except for canned anchovy, which showed a percentage close to 30% (Table 5 and Figure 2).

**Figure 3:**
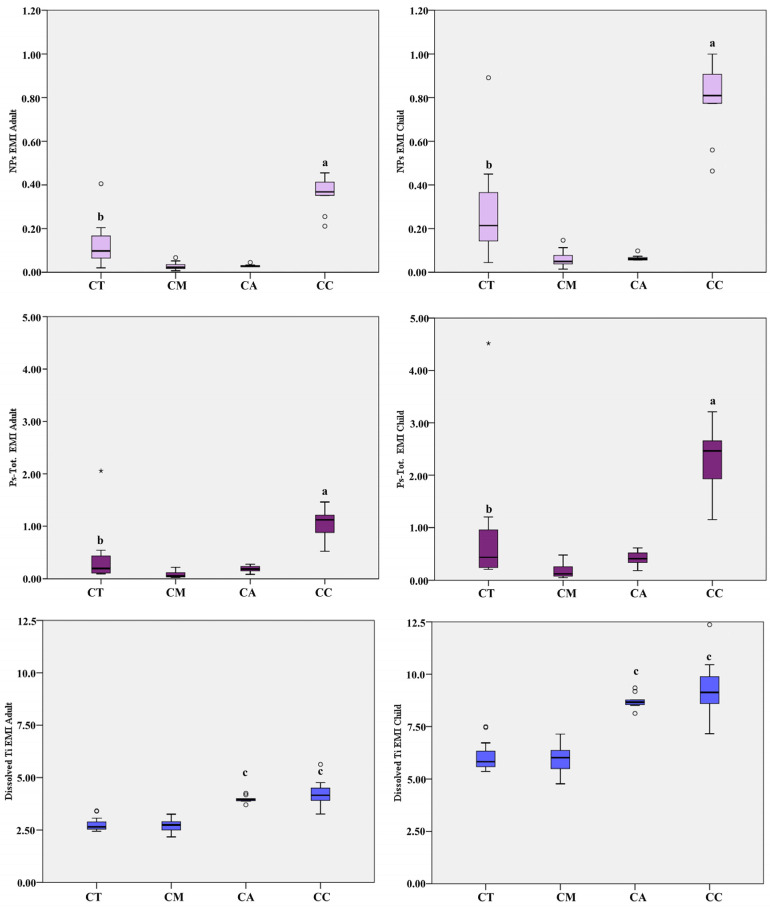
Box-plot distribution of estimated meal intake (EMI mg/kg BW) of TiO_2_-NPs, TiO_2_-Ps-Tot, and dissolved Ti concentration calculated for adults (70 years) and children (6 years). Legend: CT, canned tuna; CM, canned mackerel; CA, canned anchovy; CC, canned clam; a, *p* < 0.001 vs. all; b, *p* < 0.01 vs. CM and CA; c, *p* < 0.001 vs. CT and CM. ° Outliers values of the distribution. * Extreme values of the distribution.

Regarding the estimated meal intake, shown in Table 6 and Figure 3, bas significantly higher levels were found in children than in adults, due to the low body weight of the considered age class. According to the different seafood products, the EMI of both NPs and Ps-Tot was highest with a meal of canned clam, followed by the EMI calculated for canned tuna, significantly higher than the EMI derived from a meal of canned mackerel or canned anchovy. With regard to the dissolved Ti, the EMIs calculated for canned clam and canned anchovy were both significantly higher than the EMI calculated for the other fish species (*p* < 0.001).

## 4. Discussion

Our results are the first to provide a quantitative analysis of TiO_2_-Ps-Tot in packaged pelagic fish and to deepen findings related to bivalve mollusks. It is important to specify that, in all the products we chose, the use of the additive E171 was not indicated on the label, indicating that it was not intentionally added during food processing. Thus, the presence of titanium dioxide in particle or nanoparticle form may be attributed to contamination within the food industry, in addition to the bioaccumulation of organisms in their natural environment. As shown in Table 5 and Figure 2, our findings highlighted how the longest-lived and larger-sized organisms, such as tuna and mackerel, had a lower TiO_2_ nanoparticle diameter than anchovy, a small pelagic fish, and clams, as well as the highest percentage of NPs in the total particles. Accordingly, it seems that nanoparticles of smaller diameter have a higher potential of bioaccumulation and biomagnification along the food chain in aquatic systems. This environmental behavior of TiO_2_-NPs seems to be promoted by their poor water solubility and long-term persistence in aquatic systems [36].

Nevertheless, both TiO_2_-Ps-Tot and TiO_2_-NP concentrations were found to be significantly higher in clam, surely due to their filter-feeding behavior, making it a great biological model to test for the bioavailability of contamination at the sediment–water interface. The sedimentation capacity of TiO_2_-NPs is strictly related to the availability and characteristic of natural organic matter (NOM). NOM can act to maintain smaller particle sizes and more negative surface charges on the NPs [37], reducing the rate of sedimentation of metal oxide NPs [38,39]. As experimentally proven, the low NOM content and high ionic strength of seawater promote a high rate of sedimentation of metal oxide nanoparticles [38]. In this way, NPs are less mobile and their interaction with filter-feeders and sediment-dwelling organisms is favored [40], supporting our findings in stating that benthic mollusks are more exposed to TiO_2_-NP bioaccumulation.

The first occurrence of data related to the single-particle characterization and quantification of TiO_2_-Ps-Tot in shellfish was reported in [26,27], although the authors did not reduce the particle acquisition spectrum to the nano-fraction of 100 nm in diameter. Taboada-López et al. (2018) processed different shellfish species collected both fresh and frozen at the supermarket, by applying an ultrasound-assisted enzyme digestion and *sp*ICP-MS analysis. The levels of dissolved Ti they found in fresh clam species (0.802–1.31 mg/kg wet weight) are comparable to ours (1.31 ± 0.21 mg/kg wet weight), while they found a slightly lower particle concentration (4.16–7.56 × 10^7^ parts/g vs. 10.5 ± 1.52 × 10^7^ parts/g). Furthermore, Taboada-López et al. (2018), by analyzing the variegated scallop, found a significantly higher concentration of TiO_2_ particles and dissolved Ti in frozen samples than fresh ones, suggesting contamination by the food industry. Overall, the mean size of TiO_2_-NP diameter given for these shellfish was in the range of 60–84 nm versus a mean size of 89.8 ± 6 nm found in our packaged clams. Xu et al. (2018) also performed an enzyme digestion and *sp*ICP-MS analysis in shellfish collected from an offshore aquaculture farm. In clams, they found comparable concentrations of dissolved Ti (1.11 ± 0.35 mg/kg wet weight) and mean size of TiO_2_-NPs (70.9 ± 12.4 nm), but a lower concentration of TiO_2_-NPs (2.10 ± 0.31 × 10^7^ parts/g). Furthermore, they did not find any significant differences with the other shellfish species analyzed.

We also provided a first estimate of TiO_2_-NPs, TiO_2_-Ps-Tot, and dissolved Ti intake derived from a meal consisting of the different packaged seafood, both for adults of 70 years and children of 6 years. Results were significantly higher in children than in adults, due to the low body weight of the considered age class.

According to a carcinogenicity study with TiO_2_ (as E171) in mice and in rats, the EFSA Panel on Food Additives and Nutrient Sources added to Food (ANS) chose the lowest no observed adverse effects levels (NOAEL), which was 2250 mg TiO_2_/kg BW/day [5], but it may contain up to 3.2% of its weight in nanoparticles. For these reasons, E171 as a food additive is not considered as a nanomaterial according to the EU recommendation on the definition of a nanomaterial [5]. In our samples, we found a high occurrence of TiO_2_ in its nanoparticle structure, with the lowest in canned anchovy (32%) and the highest in canned tuna (59%); thus, we cannot refer to the established NOAEL for toxicological evaluation.

Most studies available evaluated the oral intake of total TiO_2_ and TiO_2_-NPs for TiO_2_ added during the production process in food, toothpaste, and supplements [8,11,41]. Bachler et al. (2015), by developing a physiologically based pharmacokinetic (PBPK) model, calculated the dietary intake of total titanium for the German population as between 0.5 and 1.0 mg/kg BW for all age groups except the age group “other children”, who had the highest titanium intake of all age classes (approximately 2.0 mg/kg BW). Furthermore, by assuming that 10% of the total titanium intake is in the nano-range, which is a conservative estimation according to the ratio of ionic to particulate titanium intake (1:19) and the amount of TiO_2_-NPs in E171 (approximately 11% by weight) [11], they calculated the 95th percentile of TiO_2_-NPs dietary intake as below 170 ng/g for all age groups. Moreover, Rompelberg et al. (2016) estimated the long-term intake of TiO_2_-NPs for the Dutch population as ranging from 0.19 µg/kg BW/day for the elderly to 0.55 µg/kg BW/day for 7–69-year-olds and 2.16 µg/kg BW/day for young children. The results found in the literature are significantly lower than the potential exposure calculated in this study. Nevertheless, considering that the presence of E171 as a food additive in canned fish is not expected, our results give an estimate of the dietary intake probably related to TiO_2_-NP bioaccumulation in fish and seafood species through environmental contamination. However, we cannot be sure regarding the potential total amount of TiO_2_-NPs which can be accumulated in human tissue after oral ingestion, considering that part of the compound can be altered in the gastrointestinal environment by extreme pH and co-ingested food [42], although intestinal epithelial cells are affected at a functional level [43]. Lastly, a study limitation was the inability to discern the origin of TiO_2_-NPs, i.e., natural or anthropogenic. Taking into account the numerous applications of TiO_2_-NPs, a proper improvement of the wastewater depuration process could be a step to prevent the contamination of the water bodies.

## 5. Conclusions

This study provided, for the first time, an evaluation of the estimated daily intake of TiO_2_-NPs starting from quantitatively measured data. Our findings highlighted how processed seafood, where the pigment E171 is not intentionally added, may contain amounts of TiO_2_ in its particle and nanoparticle form, as well as dissolved titanium. In particular, our results revealed that the consumption of selected types of foods could be an important route for the uptake of TiO_2_, especially for a vulnerable group such as children. Processed clam was the seafood with the highest content of TiO_2_-NPs, while, among pelagic fish species, we found progressively higher levels and smaller sizes from small to large fish. Nevertheless, seafood consumption represents only a small part of the human total diet; thus, to provide a realistic exposure assessment, it is important to carry out TiO_2_-NP characterization and quantification in a large number of food items, both processed and not, as well as where the pigment E171 is intentionally added and not, as it is not the only source of TiO_2_-NPs. This information would be a solid step toward being able to actually estimate the TiO_2_-NP dietary exposure of populations and the related health risks. The new emerging technique of *sp*ICP-MS, which is able to distinguish between the particulate and the dissolved fraction, is crucial for a better understanding of the fate and transport characteristics of the particles and ionic forms, as well as their possible (independent or synergistic) environmental and (eco) toxicological impacts.

## Figures and Tables

**Table 1:** NexION^®^ 350D inductively coupled plasma mass spectrometry (ICP-MS) instrumental condition for single particles analysis. RF, radiofrequency.

Parameter	Value
Nebulizer, flow	Meinhard, 1 mL/min
Spray chamber	Glass cyclonic
Sample uptake rate	0.26–0.28 mL/min
RF power	1600 W
Analysis mode	Standard
Quadrupole settling time	0 µs
Analyte	Ti 48.9
Dwell time	50 µs
Data acquisition time	60 s
Density	4.23 g/cm^3^
Ti mass fraction	60%

**Table 2:** NexION^®^ 350D ICP-MS instrumental condition for Total Ti in standard mode.

Parameter	Value
Nebulizer, flow	Meinhard, 0.89 mL/min
Spray chamber	Glass cyclonic
RF power	1600 W
Analogic phase voltage	−1950 V
Pulses voltage	1300 V
Discriminator threshold	12
Deflector voltage	−12 V
Analysis mode	Standard
Analyte	Ti 48.9
Internal standard	Y

**Table 3:** Descriptive statistics concerning the quantification of TiO_2_ particles (Ps-Tot) expressed as particles/mL detected with Syngistix Nano Application software after acid digestion of the samples.

Statistics	4 µg/L Ionic Ti (*n* = 6)	Canned Clam (*n* = 6)	Canned Tuna (*n* = 6)
Mean of particles/mL	564	588	653
SD	155	267	237
Min	324	267	252
Max	758	901	918
Mean + 3 SD	1029	-	-

**Table 4:** Descriptive statistics concerning the chemical characterization and quantification of total TiO_2_ particles (Ps-Tot), dissolved Ti (mg/kg), and total Ti in packaged seafood products.

**Canned Tuna**	**Most Frequent Size Ps-Tot. (nm)**	**Mean Diameter Ps-Tot. (nm)**	**Number of Ps-Tot/g.**	**Ps-Tot. mg/kg**	**Dissolved Ti ^a^ mg/kg**	**Total Ti ^b^ mg/kg**
Mean	73	104	9.77 × 10^7^	0.117	0.851	1.015
SD	11	12	6.27 × 10^7^	0.156	0.099	0.121
Min.	59	87	1.93 × 10^7^	0.029	0.752	0.857
Max.	98	126	2.86 × 10^8^	0.634	1.052	1.152
**Canned Mackerel**	**Most Frequent size Ps-Tot. (nm)**	**Mean Diameter Ps-Tot. (nm)**	**Number of Ps-Tot/g.**	**Ps-Tot. mg/kg**	**Dissolved Ti ^a^ mg/kg**	**Total Ti ^b^ mg/kg**
Mean	85	112	1.47 × 10^7^	0.025	0.835	1.154
SD	11	11	7.17 × 10^6^	0.021	0.095	0.108
Min.	69	95	8.15 × 10^6^	0.007	0.669	0.951
Max.	104	133	2.72 × 10^7^	0.068	1.003	1.185
**Canned Anchovy**	**Most Frequent size Ps-Tot. (nm)**	**Mean Diameter Ps-Tot. (nm)**	**Number of Ps-Tot/g.**	**Ps-Tot. mg/kg**	**Dissolved Ti ^a^ mg/kg**	**Total Ti ^b^ mg/kg**
Mean	113	141	1.67 × 10^7^	0.059	1.223	1.385
SD	7	8	3.12 × 10^6^	0.019	0.051	0.148
Min.	101	127	1.06 × 10^7^	0.026	1.141	0.985
Max.	122	150	2.02 × 10^7^	0.087	1.312	1.485
**Canned Clam**	**Most Frequent size Ps-Tot. (nm)**	**Mean Diameter Ps-Tot. (nm)**	**Number of Ps-Tot/g.**	**Ps-Tot. mg/kg**	**Dissolved Ti ^a^ mg/kg**	**Total Ti ^b^ mg/kg**
Mean	82	138	1.05 × 10^8^	0.326	1.313	1.458
SD	6	13	1.52 × 10^7^	0.084	0.210	0.341
Min.	75	124	7.00 × 10^7^	0.162	1.005	1.145
Max.	91	167	1.00 × 10^8^	0.451	1.735	1.915

^a^ Ultrasound-assisted alkaline digestion and *sp*ICP-MS determination. ^b^ Microwave-assisted acid digestion and ICP-MS determination in standard mode.

**Table 5:** Descriptive statistics concerning the chemical characterization and quantification of TiO_2_ nanoparticles (TiO_2_-NPs) (<100 nm) in packaged seafood products.

**Canned Tuna**	**Most Frequent Size NPs (nm)**	**Mean Diameter NPs (nm)**	**Number of NPs/g**	**NPs mg/kg**	**%NPs on Ps-Tot.**
Mean	66	72	5.26 × 10^7^	0.0380	58.5
SD	7	4	2.19 × 10^7^	0.0298	13.3
Min.	56	65	9.34 × 10^6^	0.0062	32
Max.	82	79	1.02 × 10^8^	0.1251	76
**Canned Mackerel**	**Most Frequent size NPs (nm)**	**Mean Diameter NPs (nm)**	**Number of NPs/g**	**NPs mg/Kg**	**%NPs on Ps-Tot.**
Mean	77	78	7.84 × 10^6^	0.0085	57.4
SD	11	8	2.42 × 10^6^	0.0053	12.2
Min.	48	54	5.24 × 10^6^	0.0020	39
Max.	91	86	1.23 × 10^7^	0.0206	73
**Canned Anchovy**	**Most Frequent size NPs (nm)**	**Mean Diameter NPs (nm)**	**Number of NPs/g**	**NPs mg/Kg**	**%NPs on Ps-Tot.**
Mean	93	89	5.16 × 10^6^	0.0092	31.6
SD	2	1	9.30 × 10^5^	0.0019	6.78
Min.	91	88	4.36 × 10^6^	0.0078	23
Max.	96	92	7.24 × 10^6^	0.0137	43
**Canned Clam**	**Most Frequent size NPs (nm)**	**Mean Diameter NPs (nm)**	**Number of NPs/g**	**NPs mg/Kg**	**%NPs on Ps-Tot.**
Mean	72	89	5.37 × 10^7^	0.1116	51.4
SD	5	6	8.34 × 10^6^	0.0254	5.70
Min.	63	81	4.00 × 10^7^	0.0650	41
Max.	81	97	7.00 × 10^7^	0.1403	61

**Table 6:** Descriptive statistics of estimated meal intake (EMI mg/kg BW) calculated for adults (70 years) and children (6 years) concerning the TiO_2_-NPs, TiO_2_-Ps-Tot, and dissolved Ti.

**Canned Tuna**	**EMI Adult NPs**	**EMI Child NPs**	**EMI Adult Ps-Tot.**	**EMI Child Ps-Tot.**	**EMI Adult Dissolved Ti**	**EMI Child Dissolved Ti**
Mean	0.124	0.272	0.379	0.832	2.759	6.063
SD	0.097	0.212	0.507	1.113	0.321	0.706
Min.	0.020	0.044	0.095	0.209	2.438	5.357
Max.	0.406	0.891	2.056	4.517	3.412	7.497
**Canned Mackerel**	**EMI Adult NPs**	**EMI Child NPs**	**EMI Adult Ps-Tot.**	**EMI Child Ps-Tot.**	**EMI Adult Dissolved Ti**	**EMI Child Dissolved Ti**
Mean	0.028	0.060	0.082	0.180	2.709	5.952
SD	0.017	0.038	0.066	0.146	0.307	0.674
Min.	0.007	0.014	0.021	0.046	2.171	4.769
Max.	0.067	0.147	0.219	0.482	3.253	7.147
**Canned Anchovy**	**EMI Adult NPs**	**EMI Child NPs**	**EMI Adult Ps-Tot.**	**EMI Child Ps-Tot.**	**EMI Adult Dissolved Ti**	**EMI Child Dissolved Ti**
Mean	0.030	0.066	0.191	0.419	3.967	8.716
SD	0.006	0.013	0.062	0.135	0.164	0.361
Min.	0.025	0.055	0.084	0.184	3.699	8.129
Max.	0.044	0.098	0.280	0.616	4.254	9.346
**Canned Clam**	**EMI Adult NPs**	**EMI Child NPs**	**EMI Adult Ps-Tot.**	**EMI Child Ps-Tot.**	**EMI Adult Dissolved Ti**	**EMI Child Dissolved Ti**
Mean	0.362	0.795	1.056	2.319	4.259	9.358
SD	0.082	0.181	0.273	0.600	0.681	1.496
Min.	0.211	0.464	0.524	1.152	3.259	7.162
Max.	0.455	0.999	1.462	3.211	5.628	12.36

## References

[1] Lespes G., Faucher S., Slaveykova V.I. (2020). Natural Nanoparticles, Anthropogenic Nanoparticles, Where Is the Frontier?. Front. Environ. Sci..

[2] Ziental D., Czarczynska-Goslinska B., Mlynarczyk D.T., Glowacka-Sobotta A., Stanisz B., Goslinski T., Sobotta L. (2020). Titanium Dioxide Nanoparticles: Prospects and Applications in Medicine. Nanomaterials.

[3] Boverhof D.R., David R.M. (2010). Nanomaterial characterization: Considerations and needs for hazard assessment and safety evaluation. Anal. Bioanal. Chem..

[4] Piccinno F., Gottschalk F., Seeger S., Nowack B. (2012). Industrial production quantities and uses of ten engineered nanomaterials in Europe and the world. J. Nanopart. Res..

[5] EFSA (2016). Re-evaluation of titanium dioxide (E 171) as a food additive. EFSA J..

[6] Filippini T., Tancredi S., Malagoli C., Malavolti M., Bargellini A., Vescovi L., Nicolini F., Vinceti M. (2019). Dietary Estimated Intake of Trace Elements: Risk Assessment in an Italian Population. Expo. Health.

[7] Silano V., Baviera J.M.B., Bolognesi C., Tlustos C., Loveren H.V., Vernis L., Zorn H., Castle L., Cravedi J.P., Kolf-Clauw M. (2019). Safety assessment of the substance, titanium dioxide surface treated with fluoride-modified alumina, for use in food contact materials. EFSA J..

[8] Rompelberg C., Heringa M.B., van Donkersgoed G., Drijvers J., Roos A., Westenbrink S., Peters R., van Bemmel G., Brand W., Oomen A.G. (2016). Oral intake of added titanium dioxide and its nanofraction from food products, food supplements and toothpaste by the Dutch population. Nanotoxicology.

[9] Schneider S.L., Lim H.W. (2019). A review of inorganic UV filters zinc oxide and titanium dioxide. Photodermatol. Photoimmunol. Photomed..

[10] Ruszkiewicz J.A., Pinkas A., Ferrer B., Peres T.V., Tsatsakis A., Aschner M. (2017). Neurotoxic effect of active ingredients in sunscreen products, a contemporary review. Toxicol. Rep..

[11] Weir A., Westerhoff P., Fabricius L., von Goetz N. (2012). Titanium Dioxide Nanoparticles in Food and Personal Care Products. Environ. Sci. Technol..

[12] Bostan H.B., Rezaee R., Valokala M.G., Tsarouhas K., Golokhvast K., Tsatsakis A.M., Karimi G. (2016). Cardiotoxicity of nano-particles. Life Sci..

[13] Klaine S.J., Alvarez P.J.J., Batley G.E., Fernandes T.F., Handy R.D., Lyon D.Y., Mahendra S., McLaughlin M.J., Lead J.R. (2008). Nanomaterials in the environment: Behavior, fate, bioavailability, and effects. Environ. Toxicol. Chem..

[14] Carmo T.L.L., Azevedo V.C., Siqueira P.R., Galvão T.D., Santos F.A., Martinez C.B.R., Appoloni C.R., Fernandes M.N. (2018). Mitochondria-rich cells adjustments and ionic balance in the Neotropical fish Prochilodus lineatus exposed to titanium dioxide nanoparticles. Aquat. Toxicol..

[15] Westerhoff P., Song G., Hristovski K., Kiser M.A. (2011). Occurrence and removal of titanium at full scale wastewater treatment plants: Implications for TiO_2_ nanomaterials. J. Environ. Monit..

[16] Borm P.J.A., Robbins D., Haubold S., Kuhlbusch T., Fissan H., Donaldson K., Schins R., Stone V., Kreyling W., Lademann J. (2006). The potential risks of nanomaterials: A review carried out for ECETOC. Part. Fibre Toxicol..

[17] Chen C., Marcus I.M., Waller T., Walker S.L. (2018). Comparison of filtration mechanisms of food and industrial grade TiO2 nanoparticles. Anal. Bioanal. Chem..

[18] Jovanović B. (2015). Critical review of public health regulations of titanium dioxide, a human food additive: Titanium Dioxide in Human Food. Integr. Environ. Assess. Manag..

[19] Chen Z., Han S., Zhou D., Zhou S., Jia G. (2019). Effects of oral exposure to titanium dioxide nanoparticles on gut microbiota and gut-associated metabolism in vivo. Nanoscale.

[20] Schneider T., Westermann M., Glei M. (2017). In vitro uptake and toxicity studies of metal nanoparticles and metal oxide nanoparticles in human HT29 cells. Arch. Toxicol..

[21] Chen Z., Zhou D., Zhou S., Jia G. (2019). Gender difference in hepatic toxicity of titanium dioxide nanoparticles after subchronic oral exposure in Sprague-Dawley rats. J. Appl. Toxicol..

[22] Heringa M.B., Geraets L., van Eijkeren J.C.H., Vandebriel R.J., de Jong W.H., Oomen A.G. (2016). Risk assessment of titanium dioxide nanoparticles via oral exposure, including toxicokinetic considerations. Nanotoxicology.

[23] Chen Z., Han S., Zheng P., Zhou D., Zhou S., Jia G. (2020). Effect of oral exposure to titanium dioxide nanoparticles on lipid metabolism in Sprague-Dawley rats. Nanoscale.

[24] Cao X., Han Y., Gu M., Du H., Song M., Zhu X., Ma G., Pan C., Wang W., Zhao E. (2020). Foodborne Titanium Dioxide Nanoparticles Induce Stronger Adverse Effects in Obese Mice than Non-Obese Mice: Gut Microbiota Dysbiosis, Colonic Inflammation, and Proteome Alterations. Small.

[25] Taboada-López M.V., Herbello-Hermelo P., Domínguez-González R., Bermejo-Barrera P., Moreda-Piñeiro A. (2019). Enzymatic hydrolysis as a sample pre-treatment for titanium dioxide nanoparticles assessment in surimi (crab sticks) by single particle ICP-MS. Talanta.

[26] Taboada-López M.V., Iglesias-López S., Herbello-Hermelo P., Bermejo-Barrera P., Moreda-Piñeiro A. (2018). Ultrasound assisted enzymatic hydrolysis for isolating titanium dioxide nanoparticles from bivalve mollusk before sp-ICP-MS. Anal. Chim. Acta.

[27] Xu L., Wang Z., Zhao J., Lin M., Xing B. (2020). Accumulation of metal-based nanoparticles in marine bivalve mollusks from offshore aquaculture as detected by single particle ICP-MS. Environ. Pollut..

[28] Yin C., Zhao W., Liu R., Liu R., Wang Z., Zhu L., Chen W., Liu S. (2017). TiO_2_ particles in seafood and surimi products: Attention should be paid to their exposure and uptake through foods. Chemosphere.

[29] Zhou Q., Liu L., Liu N., He B., Hu L., Wang L. (2020). Determination and characterization of metal nanoparticles in clams and oysters. Ecotoxicol. Environ. Saf..

[30] Domingo J.L., Bocio A., Falcó G., Llobet J.M. (2007). Benefits and risks of fish consumption: Part I. A quantitative analysis of the intake of omega-3 fatty acids and chemical contaminants. Toxicology.

[31] Wu S., Zhang S., Gong Y., Shi L., Zhou B. (2020). Identification and quantification of titanium nanoparticles in surface water: A case study in Lake Taihu, China. J. Hazard. Mater..

[32] Gray E.P., Coleman J.G., Bednar A.J., Kennedy A.J., Ranville J.F., Higgins C.P. (2013). Extraction and Analysis of Silver and Gold Nanoparticles from Biological Tissues Using Single Particle Inductively Coupled Plasma Mass Spectrometry. Environ. Sci. Technol..

[33] Lee S., Bi X., Reed R.B., Ranville J.F., Herckes P., Westerhoff P. (2014). Nanoparticle Size Detection Limits by Single Particle ICP-MS for 40 Elements. Environ. Sci. Technol..

[34] Copat C., Arena G., Fiore M., Ledda C., Fallico R., Sciacca S., Ferrante M. (2013). Heavy metals concentrations in fish and shellfish from eastern Mediterranean Sea: Consumption advisories. Food Chem. Toxicol..

[35] US-EPA (2000). Guidance for Assessing Chemical Contamination Data for Use in Fish Advisories—Volume II. Risk Assessment and Fish Consumption Limits EPA/823-B94-004.

[36] Gupta G.S., Kumar A., Shanker R., Dhawan A. (2016). Assessment of agglomeration, co-sedimentation and trophic transfer of titanium dioxide nanoparticles in a laboratory-scale predator-prey model system. Sci. Rep..

[37] Zhang Y., Chen Y., Westerhoff P., Crittenden J. (2009). Impact of natural organic matter and divalent cations on the stability of aqueous nanoparticles. Water Res..

[38] Keller A.A., Wang H., Zhou D., Lenihan H.S., Cherr G., Cardinale B.J., Miller R., Ji Z. (2010). Stability and Aggregation of Metal Oxide Nanoparticles in Natural Aqueous Matrices. Environ. Sci. Technol..

[39] Pettibone J.M., Cwiertny D.M., Scherer M., Grassian V.H. (2008). Adsorption of Organic Acids on TiO_2_ Nanoparticles: Effects of pH, Nanoparticle Size, and Nanoparticle Aggregation. Langmuir.

[40] Farré M., Gajda-Schrantz K., Kantiani L., Barceló D. (2009). Ecotoxicity and analysis of nanomaterials in the aquatic environment. Anal. Bioanal. Chem..

[41] Bachler G., Goetz N von Hungerbuhler K. (2015). Using physiologically based pharmacokinetic (PBPK) modeling for dietary risk assessment of titanium dioxide (TiO_2_) nanoparticles. Nanotoxicology.

[42] Walczak A.P., Kramer E., Hendriksen P.J., Helsdingen R., van der Zande M., Rietjens I.M., Bouwmeester H. (2015). In vitro gastrointestinal digestion increases the translocation of polystyrene nanoparticles in an in vitro intestinal co-culture model. Nanotoxicology.

[43] Guo Z., Martucci N.J., Moreno-Olivas F., Tako E., Mahler G.J. (2017). Titanium Dioxide Nanoparticle Ingestion Alters Nutrient Absorption in an In Vitro Model of the Small Intestine. NanoImpact.

